# Type and Quantity of Shipborne Garbage at Selected Tropical Beaches

**DOI:** 10.1155/2016/5126951

**Published:** 2016-10-12

**Authors:** Julyus-Melvin Mobilik, Teck-Yee Ling, Mohd-Lokman Husain, Ruhana Hassan

**Affiliations:** ^1^Department of Aquatic Science, Faculty of Resource Science and Technology, Universiti Malaysia Sarawak, 94300 Kota Samarahan, Sarawak, Malaysia; ^2^School of Marine and Environmental Sciences, Universiti Malaysia Terengganu, 21030 Kuala Terengganu, Terengganu, Malaysia

## Abstract

Marine debris is widely distributed at the coastal area of the global oceans; however, shipborne garbage source studies are still lacking to document the pollution in Malaysia Territorial Water. Thus, this study has adopted a standard method of beach marine debris survey at five beaches and inspected 115 vessels to assess the type and amount of debris from shipping source stranded on the beach. This study found that vessel visiting Malaysian ports observed the MARPOL 73/78 Annex V requirements; however, identified objects from shipping activity (1.3%; 2 items/km) found on the beaches indicate that there are vessels disposing of garbage illegally at sea. Therefore, there is a need to promote the use of biodegradable material and introduce environmental education to increase awareness on the vessel.

## 1. Introduction

The marine debris impact on marine environment has been the international concern and media attention around the world. Beaches across the world are polluted with marine debris, which poses an imminent threat to marine ecosystem [1–3]. Marine debris found on the beaches are derived from either land-based or ocean-based sources. Debris from run-off, deliberately dumped or blown by wind, contribute 80%, while 20% comes from vessels and offshore platforms [4–7]. In addition, the increasingly used highly visible nonbiodegradable products are illegally discharged and washed along the shoreline including large and buoyant plastic-based material [8–12]. Garbage from urban areas can become marine debris if it gets into rivers [4, 13] which eventually arrives in the sea or stranded on the beaches.

Although debris derived from ocean-based or marine sources may originate from commercial shipping, fishing vessels, recreational boats, fish farming, cruise liners, military fleets, research vessels, passenger ferries, offshore oil and gas platforms, and service vessels [6], shipborne garbage information focused on shipping activities is limited [14–17]. The widely used plastic-based material in the maritime activities especially in fishing equipment gears, ships' operation, and ships' food packaging has been reported to have adverse effects on the marine wildlife through entanglement and ingestion [18, 19]. Although accumulation of marine debris can be serious particularly in areas of high maritime traffic or ocean-based activities or circulating ocean currents [6, 20, 21], there is little information on the relationship between debris from ship and the presence of similar debris on the beaches [16, 17, 22]. Nevertheless, vessels may contribute to debris accumulation on beaches; however, the quantity of shipborne garbage trends has been manipulated or extrapolated [16, 23, 24]. To determine the estimated shipborne garbage amount is subject to vast variability including sampling method, combination of multi-point-source inputs, oceanographic influences, and the spreading of debris leading to great temporal and spatial litter loads variability in the marine environment [8, 22].

Since study on beach debris and shipborne garbage relationship in Malaysia is limited, this study can be used as a baseline for future reference for a more comprehensive data set. Therefore, this study aimed to (1) access the abundance of debris at beach and ship surveys, (2) determine objects origin collected at beach and ship surveys, and (3) investigate the relationship of shipborne garbage waste abundance stranded at the beach.

## 2. Materials and Methods

### 2.1. Beach Survey

**Figure 1:**
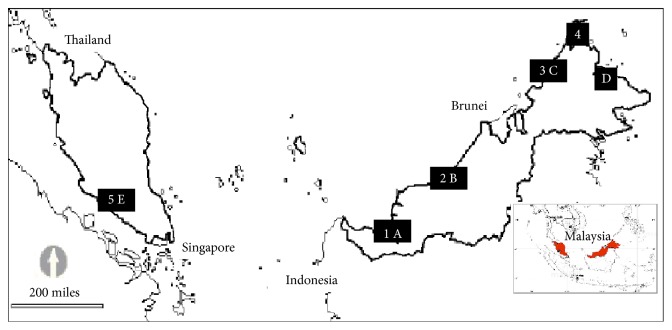
Map showing beach (numeric) and ship (alphabetic) surveys location.

Five public beaches (Table 1 and Figure 1) have been selected for beach marine debris surveys according to standing stock method [25]. All debris items other than fragments smaller than 0.25 cm^2^ within one km sampling transects were collected during the northeast monsoon (NEM), intermediate monsoon (IM), and southwest monsoon (SWM) seasons within the period of October 2012 to October 2014. After debris item collected has been identified, weighed, classified, and sorted into debris categories [26] and sources [27], each debris item was examined to identify debris items country of origin. While the debris is being sorted, the country of origin is recorded using any information still present, such as barcode, the manufacturer's name, address, and logo. To examine debris stranded on the beach and accumulated on the vessel relationship, only objects from marine source [27] were considered in the statistical analysis. Thus, marine source was further classified according to submarine source: marine ship (6 objects), marine fishing (7 objects), or marine common (8 objects). Beach debris item per km (BDI) was calculated using

(1) $$\begin{array}{c} BDI=\frac{1}{6L}\left[\;\overset{6}{\underset{i=1}{\sum }}\left(\;OI_{i}\;\right)\;\right], \end{array}$$

where BDI is total number of objects (item/km), *i* is the number of samplings conducted at each study site, *L* is the length (km) of the beach surveyed, and *OI*_*i*_ is the total number of objects collected (number,* item*).

### 2.2. Ship Survey

Five ports (Table 1 and Figure 1) have been selected to investigate shipborne objects origin on container (34 vessels), bulk carrier (46 vessels), and general cargo (35 vessels) ship types plying international route. A total of 115 vessels with 2,295 crews were involved in this study from October 2012 to October 2014. During inspection, all debris other than fragments smaller than 0.25 cm^2^ at the vessels' garbage station was examined to identify garbage item's sector or country of origin. All objects which are affixed with any information present, such as barcode, the manufacturer's name, address, and logo, were recorded. Shipborne garbage item per vessel (SGI) was calculated using

(2) $$\begin{array}{c} SGI=\frac{n_{i}}{n_{v}}, \end{array}$$

where SGI is total number of objects (item/vessel), *n*_*i*_ is the total shipborne objects (number,* item*), and *n*_*v*_ is total number of vessels sampled.

### 2.3. Data Analysis

In this study, only objects bearing labels indicating country of origin (logo, EAN (European Article Number) international barcodes, etc.) collected at the beach and on the vessel were considered in this analysis. Items that have the same label are listed and categorized as a source origin. These items were used as variables to analyze the debris relationship between debris on the beach and vessel surveys. Data were collected and then calculated using Microsoft Excel 2007 to provide information on percent composition of the objects, the highest and lowest encountered items, and possible sources identified for marine source from beach and ship surveys. For statistical analysis, *z*-test was used to analyze the distribution of normality using critical values smaller than ±1.96 (*n* < 50) with an alpha level of 0.05 for absolute *z*-scores for either skewness or kurtosis [28]. A log_10_ transformation (log_10_+1) of the data was applied for statistical analyses that did not assume a normal distribution [29]. Pearson's correlation test between BDI and SGI with number of vessels visiting study ports, vessels' clear plastic bottles (CPB), beach CPB, beach-urban proximity, and beach-port proximity was analyzed. Multiple linear regression (stepwise) model was used to identify predictor variable contributing to the abundance of SGI when correlation test indicated significant differences. All statistical comparisons were performed using SPSS version 22 package software.

## 3. Results and Discussion

### 3.1. Debris Abundance

#### 3.1.1. Beach Objects Abundance

 A total of 36 objects present at beach surveys were identified as commonly used household domestic products. The ten most abundant objects found at beach study sites that contributed 80.13% of the total debris items collected included CPB, plastic fragments, plastic food wrappers, and colored plastic bottles and they were found present at all study sites (Table 2). These objects contributed 14.48% (147 items/km), 10.25% (104 items/km), 10.24% (104 items/km), and 8.17% (83 items/km), respectively.

Table 3 shows the most objects found stranded on the study sites. Kosuhoi beach accumulated the highest mean BDI for CPB (244 items/km), plastic (others) (133 items/km), and colored plastic bottles (132 items/km), while Temasya beach accumulated the highest mean BDI for plastic fragments (165 items/km), food wrappers (120 items/km), bottle caps (101 items/km), cups (90 items/km), and cardboard cartons (54 items/km). Plastic food wrappers (160 items/km) and packaging (73 items/km) objects were highest at Pasir Pandak beach.

#### 3.1.2. Shipborne Objects Abundance

 As for ship survey, a total of 31 objects that were present at the vessel were identified as commonly used household domestic products. From the shipborne objects perspective, 62.01% objects from the plastic category were present at all vessel samples which included CPB, food wrappers, plastic fragments, colored plastic bottles, and cardboard cartons. The five most numerous objects found on the vessels which contributed 70.13% of the total shipborne items collected were CPB, plastic food wrappers, rubber (others), plastic fragments, and aluminum cans (Table 2). These objects contributed 30.36% (53 items/vessel), 11.96% (21 items/vessel), 10.74% (19 items/vessel), 10.56% (19 items/vessel), and 6.52% (11 items/vessel), respectively.

Analyzing objects abundance according to study ports, Sandakan port accumulated the highest mean SGI for CPB (72 items/vessel), aluminum cans (16 items/vessel), tin cans (6 items/vessel), and glass bottles (6 items/vessel) (Table 4). Kota Kinabalu port accumulated the highest mean SGI for rubber (others) (22 items/vessel) and plastic fragments (24 items/vessel), while Bintulu port was the highest for oil rags (13 items/vessel) and cardboard cartons (10 items/vessel). Plastic food wrappers and colored plastic bottles objects were highest at Kuching and Klang ports at 26 items/vessel and 11 items/vessel, respectively.

The amount of shipborne garbage accumulated in this study indicates the need for monitoring of waste entry into the vessel. The practice of accepting provision goods from a ship chandler without considering the waste generated from the provision packaging may result in continuous illegal discharge into the sea. Therefore, it is necessary to review garbage management plan (GMP) to consider generated waste from provision goods received. In promoting such effort, port authorities may introduce “no plastic day” campaign to create awareness on the effect of shipborne objects when illegally discharged into sea. Subsequently, these minor changes could contribute a greater impact towards the marine environment.

#### 3.1.3. Analysis of Marine Source Debris Found at Beach Survey

From 21 objects identified as marine source, 62% objects were present at all study sites amounting to 3,536 items including foam packaging (51 items/km), cigarette lighters (22 items/km), foam insulation (10 items/km), plastic oil bottles (10 items/km), and buckets (6 items/km). Analyzing each submarine source, marine-common source accumulated the highest items found on the beaches at 87.02% (3,977 items or 103 items/km), while marine-ship source presented the lowest items found on the beach at 2.38% (84 items or 3 items/km). Objects associated with marine-ship source present on the beach include pallet wrappers (79 items), gloves (3 items), and steel drums (2 items). Nevertheless, objects in marine-common source are of particular concern as these objects were found abundantly at all beach study sites. Objects associated with marine-common source include CPB, Styrofoam cups and plates, colored plastic bottles, plastic bottle caps, and footwear. These objects can derive from commercial vessels, domestic vessel, fishing vessel, platform, or urban areas.

Despite public concern on marine debris pollution at coastal area from marine source, little attention has been given to addressing debris from marine-ship source. The growing number of vessels en route through Malacca Straits [30–32] indicates that the amount of shipborne garbage accumulated on the vessel increases and the possibilities to illegally discharge shipborne garbage within MTW will deteriorate the marine environment. The situation may be worsened as studies on accumulation of marine debris concentrated in specific regions [33–36]; identified debris are being transported by wind and current distribution is located far from accumulation region.

Although this study is unable to quantify the amount of shipborne garbage discharged illegally, garbage disposal within 12 nmi is of particular concern as garbage discharge may be trapped by near shore current systems and transported through longshore drift current to coastal areas [37]. In addition, debris from land-based source including run-off and rubbish thrown into rivers [22, 27, 38, 39] may also be transported by longshore drift currents to coastal beach areas [37]. Therefore, the continued use of plastic-based products should be replaced with biodegradable material while cultivating environmental awareness to renew public attitude to appreciate the marine environment.

#### 3.1.4. Beach and Shipborne Object Abundance Relationship

Table 5 shows total items of marine source identified during beach survey and on the vessel. Analyzing objects found at the beach shows Kosuhoi beach accumulated the highest mean BDI (marine source) at 224 items/km, while Temasya beach accumulates the lowest at 69 items/km. As for mean CPB, Kosuhoi beach accumulated the highest at 244 items/km, as compared to Saujana beach at 45 items/km. Ship survey results showed Kota Kinabalu port accumulated the highest mean SGI as compared to Kuching port at 198 items/vessel and 163 items/vessel, respectively. As for CPB abundance, vessel visiting Sandakan and Kuching ports accumulated the highest and lowest CPB items at 72 items/vessel and 42 items/vessel, respectively.

Correlation analysis results show mean BDI (marine source) is significantly correlated (*p* < 0.05, *z* <±1.96, and *n* = 5) with urban proximity (*r* = 0.89 and *p* = 0.05), while mean SGI showed no evidence of significant correlation (*p* > 0.05). Multiple linear regression results (*R* ^2^ = 0.78 and *p* < 0.05) show that urban proximity (*β* = 0.89, *t* = 3.30, and *p* = 0.05) is a significant factor in determining BDI (BDI = 73.30 − 0.59*∗*(urban  proximity)).

Studies on floating marine debris trajectories [33–36] show wind distribution and current effect determines the amount of debris accumulations. The analyzed wind data collected by Malaysia Meteorology Department shows the wind pattern movement in the study area was in accordance with the monsoon wind circulation in unprotected beach areas at Kosuhoi and Saujana beaches. The wind pattern movement at Pasir Pandak, Temasya, and Tg. Aru beaches was erratic and could be due to the influence of hilly topography. This study found the amount of debris items was more abundant during SWM season when compared to NEM and IM seasons. Kubota et al. [35] explain that it is probable that steady wind affects debris movement at sea. Therefore, debris accumulation at the study sites may have been influenced by Ekman drift during monsoon winds.

Studies have suggested Relative Exposure Index (REI) as a possible indicator to determine marine debris accumulation, besides providing a summary of the wind/wave exposure on the beach [9, 40–42]. This relates in a number of factors to debris accumulation on the beach, including local and offshore currents, slope, beach length, tidal range, and prevailing wind. Since marine debris items are light-weighted and can travel long distances, debris deposition at the study site may be greater at higher REI value during NEM season which receives current speed of 1 m/s at 3.5 m wave height [43] and wind speed of 10 m/s [44]. However, this study found mean BDI was higher during SWM season. This finding was the opposite to the result from Silva-Cavalcanti et al. [45], Golik and Gertner [46], and Vauk and Schrey [16]. This provides indication that debris accumulation may derive from irresponsible beach-goers that do not dispose of their garbage in a civilized manner [47, 48]. The other possible explanation is the effect of longshore drift [37, 49], since coastal villages are adjacent to the beaches. Therefore, comprehensive and long-term seasonal debris trends monitoring along Malaysian coastline is eminent to identify debris abundance relationship against season, topography, wind, and wave exposures.

Analyzing Ekman currents, the debris movement is deflected left with an angle of 45° between the surface wind vectors and the Ekman current vectors. According to Wyrtki [50], the current movement patterns concentration during NEM and SWM seasons is stronger and towards the Peninsular Malaysia coastal area, whereas the current movement patterns in Sabah and Sarawak are weaker. This current movement forms a small circulation anticlockwise pattern movement during NEM but a clockwise one during SWM [43]. Thus, garbage accumulation may focus on the circulation pattern, which may be located between the Peninsula and Sabah/Sarawak water body. Therefore, the fate of shipborne floating garbage may remain in the marine environment if the garbage is illegally discharged beyond 100 nmi. This may explain the insignificant amount of marine-ship sources objects found stranded at the beach study sites.

### 3.2. Debris Origin

#### 3.2.1. Beach Debris Labeled Objects

**Figure 2:**
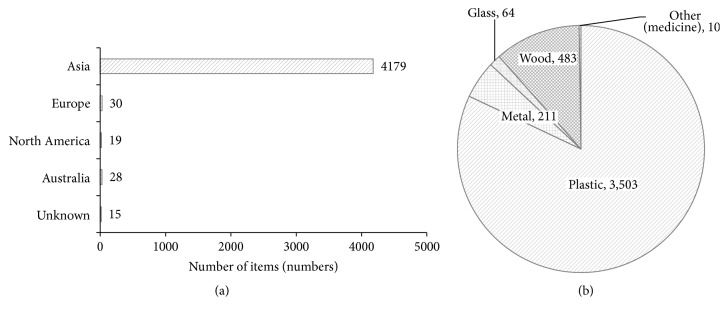
Total labeled items (number,* items*) found at beach surveys according to (a) continents and (b) debris category.

This study has identified 9.26% (4,271 items) of the total debris items found at the beach were still affixed with labels of origin. The debris items can be identified as originating from 29 countries representing six continents. The highest identified debris items were from Asia continent representing 97.86% (4,179 items) of the labeled items found at the beaches (Figure 2(a)). Analyzing debris labeled items country of origin showed 81.92% of the total labeled items originated from Malaysia (or local), followed by Indonesia (6.13%), Singapore (2.58%), Vietnam (1.64%), Thailand (1.64%), and other countries (6.09%). The abundance of labeled objects according to debris categories shows plastic category represents the highest objects accumulated at 3,503 items (82.02%), followed by wood (483 items; 11.31%), metal (211 items; 4.94%), and glass (64 items; 1.50%) categories (Figure 2(b)). The five highest objects with affixed label indicating the country of origin representing 94.97% of the total labeled objects found were CPB (43.76%), plastic food wrappers (22.24%), colored plastic bottles (14.91%), cardboard cartons (11.31%), and aluminum cans (2.74%).

Analyzing debris origin according to study sites, Kosuhoi beach accumulated the most items from local (1,122 items/km) and foreign (241 items/km) origins, respectively, whereas Saujana accumulated the lowest items for both local (37 items/km) and foreign (10 items/km) origins (Table 6).

#### 3.2.2. Shipborne Garbage Labeled Objects

**Figure 3:**
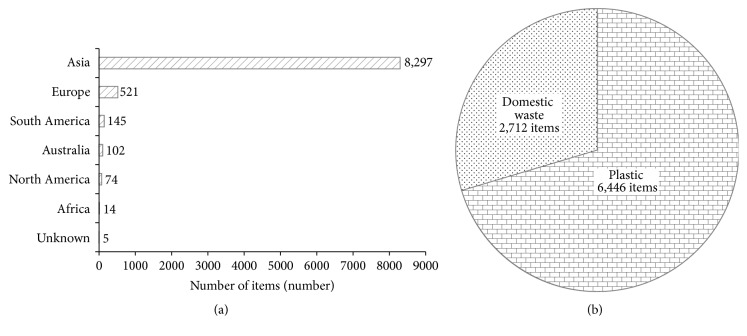
Total labeled items (number,* items*) found at ship surveys according to (a) continents and (b) debris category.

Analyzing shipborne garbage origin, 43.83% (9,158 items) were still affixed with labels indicating country of origin. Objects originating from 42 countries showed that Asia continent represents 90.60% (8,297 items) of the labeled items found on the vessel surveyed (Figure 3(a)). The labeled items indicating country of origin can be identified from Vietnam (37.07%), followed by China (19.02%), Malaysia (14.02%), Thailand (5.11%), Singapore (4.85%), and other countries (19.93%).

The abundance of labeled objects was from plastic and domestic waste categories, whereby they accumulated 6,446 items (70.39%) and 2,712 items (29.61%), respectively (Figure 3(b)). The five highest objects with affixed label indicating the country of origin representing 97.36% of the total shipborne items found were CPB (49.31%), aluminum cans (26.94%), plastic food wrappers (16.66%), cardboard cartons (3.11%), and glass bottles (1.33%).

Analyzing garbage origin according to study ports, Kuching port accumulated the highest objects for local (13 items/vessel) and foreign (130 items/vessel) origins, whereas Bintulu and Klang ports accumulated the lowest objects for local (7 items/vessel) and foreign (42 items/vessel) origins (Table 7).

As a result of rising living standard, wastes generated are becoming a crisis especially in a confined area such as on the vessel. Thus, the amount of shipborne garbage may increase on the vessel if garbage production continued in an unsustainable manner. This study found plastic-based materials such as CPB and food wrappers are discharged illegally at sea. These may contribute to the destruction of marine wildlife [51–53]. Therefore, it is essential that every crew member take responsibility for waste produced on the vessel by practicing reduction, recycling, and reuse.

Studies [23, 54, 55] have shown garbage processing equipment can facilitate vessels by reducing shipborne garbage volume to a manageable size to store on the vessels before sending to port for disposal. Depending on the type of ship, area of operation, vessels voyage duration, and number of crews, installing garbage processing equipment can be costly. Nevertheless, innovative and portable garbage processing equipment which is available in the market can be considered such as the Smart Ash Portable Waste Incinerator, Plasma Arc Waste Destruction System, and manual trash compactor which can process all types of waste generated on the vessel and reduce waste volume to between 3% and 25%. Therefore, ship owners have a wider range of options to improve and initiate commitment by investing in affordable garbage processing equipment which ultimately contributes to environmental conservation.

### 3.3. Beach and Ship Survey Debris Origin Relationship

#### 3.3.1. Debris Origin according to Objects

Table 8 shows identified objects collected at ship sampling associated with objects found during beach survey. The result shows 14 objects were identified to be present at beach and ship surveys amounting to 15,648 items. Local origin items showed a higher amount found on the beaches, whereas foreign origin items were found abundant on the vessels. The highest local origin items accumulated were CPB (2,556 items or 85 items/km), food wrappers (960 items or 32 items/km), and cardboard cartons (646 items or 22 items/km). Foreign origin items were CPB (3,591 items or 31 items/vessel), aluminum cans (2,383 items or 21 items/vessel), and food wrappers (1,336 items or 12 items/vessel).

The amount of debris items stranded on beach found in this study may have been abandoned and discharged from urban area or rubbish discarded by beach visitors. However, the presence of foreign origin objects found on the beach is of particular concern. This study found that 82% of shipborne garbage items were from local origin suggesting these vessels obtain food supply at Malaysian ports. Analyzing labeled objects shows foreign origin items dominate the abundance of objects found on the vessel including CPB (3,591 items or 31 items/vessel), aluminum cans (2,383 items or 21 items/vessel), and food wrappers (1,336 items or 12 items/vessel). In addition, 63.86% garbage items that were accumulated on the vessel could also be found on the beaches including CPB, food wrappers, rubber, and plastic fragments. However, these objects can be associated with household domestic products and may have been from urban areas. Thus, it is difficult to make a conclusion that objects originating from local origin present at the beaches did come from shipping activities. Nevertheless, there is a possibility that these objects are illegally discharged at sea given the amounts of CPB found at the beach which are adequate to make the assumption. These plastic-based items were reported to pose serious threat to turtle [56], seabirds [57], and marine biodiversity [58–60] when illegally discharged at sea. In addition, they could act as a means of transportation for invasion of marine organisms [53, 61].

Malaysia aspires to become a fully developed nation by the year 2020. Thus, this implies more vessels will be visiting Malaysian ports and using Malacca Straits as transit or innocent passage. Considering the number of vessels using Malacca Straits in year 2014 [30], the estimated total number of 95,000 vessels is expected to use Malacca Straits in year 2020. This will translate to an amount of 5,217 MT of shipborne garbage with a staggering 12.4 million of garbage items on the vessels. This study found garbage contractors engaged to collect shipborne garbage and charged between USD 200 and USD 500 for shipborne garbage collection services since the study ports were not equipped with reception facilities for receiving shipborne garbage. The high cost incurred for the handling and disposal of waste by garbage contractors can deter vessels from sending shipborne garbage to ports [62, 63]. Thus, this will aggravate illegal discharge practices from vessels navigating within MTW, eventually, magnifying the amount of garbage items in the marine environment. Therefore, there is a need to develop preventive strategies to ensure illegal discharge practices from vessel navigating within MTW are totally eliminated.

#### 3.3.2. Debris Origin according to Objects EAN International Barcodes

Table 9 shows items identified with EAN international barcode found at beach and ship survey. The result shows that five objects identified having the same EAN international barcode affix on the product were present at beach and ship surveys amounting to 2,447 items. Although there was presence of local origin objects found on the vessel with the same EAN labeled found on the beach, it is difficult to distinguish whether these objects found on the beach were originated from the vessels. However, objects of foreign origin are of particular concern. A total of 83 foreign origin items with the same EAN labeled affix found on the beach were present on the vessels including CPB (81 items) and aluminum cans (2 items) (Table 9).

From correlation analysis results, mean log_10_ beach (foreign item) is significantly correlated (*p* < 0.01, *z* <±1.96, and *n* = 5) with log_10_ ship (foreign item) (*r* = 0.98 and *p* = 0.00), whereas mean log_10_ beach (local item) showed insufficient evidence to conclude significant correlation (*p* > 0.05) against mean ship (local item). Multiple linear regression results (*R* ^2^ = 0.97 and *p* < 0.01) show log_10_ ship (foreign item) (*β* = −0.94, *t* = −4.87, and *p* = 0.02) has a strong relationship with log_10_ beach (foreign item) abundance at the beaches (log_10_ beach (foreign  item)) = 0.610*∗*(log_10_  ship  (foreign  item)).

This study demonstrates that the amount of marine debris from shipping activity found on the beach indicates crew members may have discharged shipborne garbage illegally. The factor responsible for the presence of shipborne garbage could be attributed to the attitude and behavior of an individual [48, 64, 65]. In the advent of the revise MARPOL 73/78, illegal shipborne garbage discharge practice requires stern preventive measures. Other studies [63, 66] suggested that vessels operations on a tight schedule and probability not to be detected are among the reasons illegal discharge is still being practiced.

This study has identified CPB as the most abundant objects found on the beach originating from neighboring countries. Statistical analysis results show CPB (*R* ^2^ = 0.99) may be used as an indicator to determine shipborne garbage abundance on the vessels. Figures calculated for shipborne objects abundance on container, bulk carrier, and general cargo vessels during this study period had given approximately 16.52 items/vessel for every 100 CPB of foreign origin objects found on the beach. Therefore, there is a need to conduct a detailed PSC inspection on the vessels registered with neighboring countries. In addition, the PSC inspection outcome should be communicated to the vessels' country registrar office in hope that preventive measures can be introduced on these neighboring countries registered vessels.

## 4. Conclusion

Findings in this study have identified 14 labeled objects that were present at the beach and ship surveys, representing 23% of the total debris collected originating from 75 countries. Substantially higher amounts of objects found were CPB, food wrappers, and cardboard cartons. This is expected as these objects are daily consumer goods which can be easily thrown directly on the streets, in rivers, on beaches, or into the sea. Although this is a common practice especially in urban area, from shipping activities perspective, it is totally prohibited according to MARPOL 73/78. Nevertheless, objects stranded on the beaches which can be attributed to shipping activities are of utmost concern. Although the amount of objects from shipping activity (1.3%; 2 items/km) found on the beaches was low, it indicates there are vessels disposing of garbage illegally at sea. The strong correlation (*r* = 0.98 and *p* = 0.00) between foreign origin items stranded on the beach and found on the vessel indicates CPB can be used as an indicator to estimate shipborne garbage. The use of biodegradable packaging material can be an alternative to reduce environmental pollution problem. However, it is necessary to introduce environmental education and encourage garbage disposal in a responsible and sustainable approach on the vessel.

## Figures and Tables

**Table 1:** Location of beach and port surveys.

Beach, (a)		Nearest port, (b)		Distance (a)-(b)	
Site	Lat/long	Site	Lat/long	Road (km)	Sea (km)
(1) Pasir Pandak	Start point: 01°41′30.0′′N, 110°18′11.1′′E End point: 01°41′38.9′′N, 110°18′27.8′′E	(A) Kuching	1°33′12.8′′N, 110°23′47.1′′E	22.9	57.6
(2) Temasya	Start point: 03°12′51.5′′N, 113°02′59.9′′E End point: 03°12′37.3′′N, 113°02′47.0′′E	(B) Bintulu	3°15′52.2′′N, 113°04′26.0′′E	18.2	2.8
(3) Tg. Aru	Start point: 05°56′4.1′′N, 116°2′48.6′′E End point: 05°56′29.3′′N, 116°2′47.5′′E	(C) Kota Kinabalu	5°59′42.3′′N, 116°04′59.6′′E	13.7	8.9
(4) Kosuhoi	Start point: 07°1′24.3′′N, 116°44′39.1′′E End point: 07°1′53.4′′N, 116°44′47.4′′E	(D) Sandakan	5°50′08.2′′N, 118°06′08.5′′E	422.3	235.3
(5) Saujana	Start point: 02°29′51.3′′N, 101°50′16.2′′E End point: 02°30′9.1′′N, 101°50′8.2′′E	(E) Klang	2°57′12.8′′N, 101°18′38.5′′E	165.3	74.9

**Table 2:** Total items (number, *item*) accumulated during beach and ship surveys.

Rank	Beach survey			Ship survey		
	Objects	Total items	Total (%)	Objects	Total items	Total (%)
1	Clear plastic bottles	4,407	14.48	Clear plastic bottles	6,148	30.36
2	Plastic fragments	3,121	10.25	Food wrapper	2,421	11.96
3	Plastic food wrapper	3,116	10.24	Rubber (others)	2,174	10.74
4	Colored plastic bottles	2,488	8.17	Plastic fragments	2,139	10.56
5	Plastic (others)	2,422	7.96	Aluminum cans	1,320	6.52
6	Cups	2,099	6.90	Oil rags	1,124	5.55
7	Bottle caps	1,986	6.52	Colored plastic bottles	984	4.86
8	Food wrappers	1,966	6.46	Cardboard cartons	865	4.27
9	Packaging	1,540	5.06	Tin cans	522	2.58
10	Cardboard cartons	1,246	4.09	Glass bottles	511	2.52

**Table 3:** Ten most numerous objects (item/km) found and BDI at the study sites.

Objects	Beach study sites				
	Pasir Pandak	Temasya	Tg. Aru	Kosuhoi	Saujana
Clear plastic bottles	189	118	139	244	45
Plastic fragments	66	84	165	93	113
Plastic food wrapper	160	108	128	61	64
Colored plastic bottles	130	58	85	132	8
Plastic (others)	68	33	70	133	99
Cups	52	72	90	64	71
Bottle caps	48	57	101	77	48
Food wrappers	36	59	120	56	58
Packaging	73	25	38	83	39
Cardboard cartons	30	41	54	50	33

BDI (item/km)	657	790	1,208	1,263	731

**Table 4:** Ten most numerous objects (item/vessel) found and SGI at the study ports.

Objects	Study port				
	Kuching	Bintulu	Kota Kinabalu	Sandakan	Klang
Clear plastic bottles	42	51	56	72	55
Plastic food wrapper	26	22	21	19	18
Rubber (others)	16	22	22	15	19
Plastic fragments	23	12	24	18	16
Aluminum cans	13	10	11	16	10
Oil rags	9	13	12	9	7
Colored plastic bottles	6	5	9	9	11
Cardboard cartons	7	10	8	9	6
Tin cans	4	4	4	6	5
Glass bottles	3	4	4	6	5

SGI (item/vessel)	163	168	198	194	171

**Table 5:** Mean BDI (marine source) (item/km), SGI (item/vessel), and CPB found during beach and ship surveys.

Study sites/study ports	Beach survey		Ship survey	
	BDI (marine source)	CPB	SGI	CPB
Pasir Pandak/Kuching	108	189	163	42
Temasya/Bintulu	69	118	168	51
Tg. Aru/Kota Kinabalu	115	139	198	56
Kosuhoi/Sandakan	224	244	194	72
Saujana/Klang	74	45	171	55

**Table 6:** Total items for objects (item/km) origin found during beach survey.

Objects	Pasir Pandak		Temasya		Tg. Aru		Kosuhoi		Saujana	
	L	F	L	F	L	F	L	F	L	F
Clear plastic bottles	31	7	63	5	65	1	119	9	10	2
Food wrappers	18	6	20	6	40	13	29	10	15	3
Cardboard carton	9	4	22	4	23	3	7	1	5	3
Colored plastic bottles	10	5	11	9	18	6	25	19	2	2
Aluminum cans	4	2	1	1	5	1	1	0	5	1
Aerosol cans	1	0	1	1	7	0	4	0	0	0
Glass bottles	2	0	2	0	4	2	0	0	1	0
Plastic oil bottles	0	0	1	1	0	0	2	0	0	0
Tin cans	1	0	0	0	1	0	0	0	0	0
Medicine	0	1	0	0	0	0	0	1	0	0
Product wrapper	0	0	0	0	1	0	0	0	0	0
Metal (others)	0	0	0	0	0	0	0	0	0	0
Plastic (others)	0	0	0	0	0	0	0	0	0	0
Plastic container	0	0	0	0	0	0	0	0	0	0

Total	75	26	120	27	164	26	187	40	37	10

L: local origin (or Malaysia) and F: foreign origin.

**Table 7:** Total objects (item/vessel) origins found at ship survey.

Objects	Kuching		Bintulu		Kota Kinabalu		Sandakan		Klang	
	L	F	L	F	L	F	L	F	L	F
Clear plastic bottles	9	26	4	35	10	30	5	44	10	29
Aluminum cans	1	77	0	5	0	8	2	7	1	3
Food wrappers	2	21	2	9	2	12	1	10	1	7
Cardboard cartons	1	2	1	2	0	4	0	2	0	2
Glass bottles	0	0	0	2	0	1	0	2	0	1
Tin cans	0	0	0	0	0	1	0	2	0	1
Product wrappers	0	3	0	0	0	0	0	0	0	0
Colored plastic bottles	0	1	0	0	0	0	0	0	0	0
Aerosol cans	0	0	0	0	0	1	0	0	0	0
Plastic oil bottles	0	0	0	0	0	0	0	0	0	0

Total	13	130	7	53	12	56	9	66	12	42

L: local origin (or Malaysia) and F: foreign origin.

**Table 8:** Number of objects (number, *items*) origins found at beach and ship surveys.

Objects	Beach survey		Ship survey	
	Local	Foreign	Local	Foreign
Clear plastic bottles	1,727	142	925	3,591
Food wrappers	729	221	189	1,336
Aluminum cans	397	240	84	2,383
Cardboard cartons	393	90	45	240
Colored plastic bottles	86	31	2	40
Glass bottles	68	11	11	111
Product wrapper	48	16	0	76
Tin cans	20	5	21	91
Aerosol cans	13	2	0	11
Plastic oil bottles	12	5	0	2
Medicine	2	8	0	0
Metal (others)	2	2	0	0
Plastic (others)	0	1	0	0
Plastic container	3,497	774	0	0

Total	1,727	142	1,277	7,881

**Table 9:** Total objects (number, *items*) according to EAN international barcodes found at beach and ship surveys.

Objects	Beach survey		Ship survey	
	Local	Foreign	Local	Foreign
Clear plastic bottles	342	81	644	832
Aluminum cans	42	2	222	18
Food wrappers	94	0	80	0
Colored plastic bottles	46	0	2	0
Cardboard cartons	27	0	15	0

Total	551	83	963	850

## References

[1] Oigman-Pszczol S. S., Creed J. C. (2007). Quantification and classification of marine litter on beaches along Armação dos Búzios, Rio de Janeiro, Brazil. *Journal of Coastal Research*.

[2] Thompson R. C., Olson Y., Mitchell R. P. (2004). Lost at sea: where is all the plastic?. *Science*.

[3] Derraik J. G. B. (2002). The pollution of the marine environment by plastic debris: a review. *Marine Pollution Bulletin*.

[4] Joint Group of Experts on the Scientific Aspects of Marine Environmental Protection (2009). Polution in the open ocean: a review of assesment and related studies. *GESAMP Reports & Studies*.

[5] United Nations Environment Programme (2006). *The State of the Marine Environment—Trends and Processes*.

[6] Sheavly S. B. (2005). *Marine Debris—An Overview of a Critical Issue for Our Oceans*.

[7] US Commission on Ocean Policy (2004). *An Ocean Blueprint for the 21st Century (Final Report)*.

[8] Portz L., Manzolli R. P., Ivar do Sul J. A. (2011). Marine debris on Rio Grande do Sul north coast, Brazil: spatial and temporal patterns. *Journal of Integrated Coastal Zone Management*.

[9] Walker T. R., Grant J., Archambault M.-C. (2006). Accumulation of marine debris on an intertidal beach in an urban park (Halifax Harbour, Nova Scotia). *Water Quality Research Journal of Canada*.

[10] Fujieda S., Sasaki K. (2005). Stranded debris of foamed plastic on the coast of Eta Island and Kurahashi Island in Hiroshima Bay. *Nippon Suisan Gakkaishi*.

[11] Edyvane K. S., Dalgetty A., Hone P. W., Higham J. S., Wace N. M. (2004). Long-term marine litter monitoring in the remote Great Australian Bight, South Australia. *Marine Pollution Bulletin*.

[12] Convey P., Barnes D. K. A., Morton A. (2002). Debris accumulation on oceanic island shores of the Scotia Arc, Antarctica. *Polar Biology*.

[13] Sheavly S. B. (2007). *National Marine Debris Monitoring Program: Final Program Report, Data Analysis and Summary*.

[14] Jambeck J. R., Geyer R., Wilcox C. (2015). Plastic waste inputs from land into the ocean. *Science*.

[15] Sarinas B. G. S., Docto D. O., Dumaicos M. B., Flores J. R. P. (2013). Solid waste management: compliance, practices, destination and impact among merchant vessels docking in iloilo ports, philippines. *Journal of Maritime Research*.

[16] Vauk G. J. M., Schrey E. (1987). Litter pollution from ships in the German Bight. *Marine Pollution Bulletin*.

[17] Horsman P. V. (1982). The amount of garbage pollution from merchant ships. *Marine Pollution Bulletin*.

[18] Cho D.-O. (2009). The incentive program for fishermen to collect marine debris in Korea. *Marine Pollution Bulletin*.

[19] Palabiyik H. (2003). Waste management planning for ship generated waste. *Journal of Naval Science and Engineering*.

[20] Hidalgo-Ruz V., Thiel M. (2013). Distribution and abundance of small plastic debris on beaches in the SE Pacific (Chile): a study supported by a citizen science project. *Marine Environmental Research*.

[21] Walker T. R., Reid K., Arnould J. P. Y., Croxall J. P. (1997). Marine debris surveys at Bird Island, South Georgia 1990–1995. *Marine Pollution Bulletin*.

[22] Ryan P. G., Moore C. J., van Franeker J. A., Moloney C. L. (2009). Monitoring the abundance of plastic debris in the marine environment. *Philosophical Transactions of the Royal Society B: Biological Sciences*.

[23] Zuin S., Belac E., Marzi B. (2009). Life cycle assessment of ship-generated waste management of Luka Koper. *Waste Management*.

[24] Allsopp M., Walters A., Santillo D., Johnston P. Plastic Debris in the World's Oceans. http://www.greenpeace.org/international/en/publications/reports/plastic_ocean_report.

[25] Cheshire A., Adler E., Barbière J. (2009). UNEP/IOC guidelines on survey and monitoring of marine litter. *UNEP Regional Seas Reports and Studies*.

[26] Ribic C. A., Dixon T. R., Vining I. (1992). Marine Debris Survey Manual. *NOAA Techincal Report*.

[27] Ribic C. A. (1998). Use of indicator items to monitor marine debris on a New Jersey beach from 1991 to 1996. *Marine Pollution Bulletin*.

[28] Kim H. Y. (2013). Statistical notes for clinical researchers: assessing normal distribution (2) using skewness and kurtosis. *Restorative Dentistry & Endodontics*.

[29] Ribic C. A., Sheavly S. B., Rugg D. J., Erdmann E. S. (2010). Trends and drivers of marine debris on the Atlantic coast of the United States 1997–2007. *Marine Pollution Bulletin*.

[30] Marine Department Malaysia Straits of Malacca: Annual traffic volume, 2000–2014. http://www.marine.gov.my/jlmeng/pic/article/service/statistik/BKP/2014/NUMBERS_OF_SHIPS_REPORTING_UNDER_STRAITREP_UNTIL_MEI2014.pdf.

[31] Rusli M. H. M. (2012). The application of transit passage regime in straits used for international navigation: a study of the Straits of Malacca and Singapore. *Asian Politics & Policy*.

[32] Khalid N. (2006). *Port Privatization in the Context of a Developing Nation: The Malaysia Experience*.

[33] Martinez E., Maamaatuaiahutapu K., Taillandier V. (2009). Floating marine debris surface drift: convergence and accumulation toward the South Pacific subtropical gyre. *Marine Pollution Bulletin*.

[34] Pichel W. G., Churnside J. H., Veenstra T. S. (2007). Marine debris collects within the North Pacific Subtropical Convergence Zone. *Marine Pollution Bulletin*.

[35] Kubota M., Takayama K., Namimoto D. (2005). Pleading for the use of biodegradable polymers in favor of marine environments and to avoid an asbestos-like problem for the future. *Applied Microbiology and Biotechnology*.

[36] Thiel M., Hinojosa I. A., Vásquez N., Macaya E. (2003). Floating marine debris in coastal waters of the SE-Pacific (Chile). *Marine Pollution Bulletin*.

[37] Sonu C. J., McCloy J. M., Mcarthur D. S. (1966). Longshore currents and nearshore topographies. *Coastal Engineering Proceedings*.

[38] Coe J. M., Rogers D. B. (1997). *Marine Debris: Sources, Impacts and Solutions*.

[39] Dixon T. R., Dixon T. J. (1981). Marine litter surveillance. *Marine Pollution Bulletin*.

[40] Garcon J. S., Grech A., Moloney J., Hamann M. (2010). Relative exposure index: an important factor in sea turtle nesting distribution. *Aquatic Conservation: Marine and Freshwater Ecosystems*.

[41] Rodil I. F., Lastra M. (2004). Environmental factors affecting benthic macrofauna along a gradient of intermediate sandy beaches in northern Spain. *Estuarine, Coastal and Shelf Science*.

[42] Keddy P. A. (1984). Quantifying a within-lake gradient of wave energy in Gillfillan Lake, Nova Scotia. *Canadian Journal of Botany*.

[43] Akhir M. F. M. (2012). Surface circulation and temperature distribution of southern South China sea from global ocean model (OCCAM). *Sains Malaysiana*.

[44] Malaysian Meteorological Department (2010). *Climate Information*.

[45] Silva-Cavalcanti J. S., Barbosa de Araújo M. C., Ferreira da Costa M. (2009). Plastic litter on an urban beach—a case study in Brazil. *Waste Management and Research*.

[46] Golik A., Gertner Y. (1992). Litter on the israeli coastline. *Marine Environmental Research*.

[47] Abdullah M. A., Ali N., Aznie R., Rose C., Fuad M., Jali M. (2012). Industri pelancongan dan alam sekitar di Port Dickson: menyorot titik keseimbangan antara permintaan dan penawaran. *Malaysia Journal of Society and Space*.

[48] Slavin C., Grage A., Campbell M. L. (2012). Linking social drivers of marine debris with actual marine debris on beaches. *Marine Pollution Bulletin*.

[49] Taffs K. H., Cullen M. C. (2005). The distribution and abundance of beach debris on isolated beaches of northern New South Wales, Australia. *Australasian Journal of Environmental Management*.

[50] Wyrtki K. (1961). Physical oceanography of the Southeast Asian waters. *NAGA Report*.

[51] Baulch S., Perry C. A sea of plastic: evaluating the impacts of marine debris on cetaceans. https://www.researchgate.net/file.PostFileLoader.html?id=523ff97fcf57d74e7d043cb9&assetKey=AS%3A272142459965441%401441895226384.

[52] Bellwood D. R., Hughes T. R., Folke C., Nyström M. (2004). Confronting the coral reef crisis. *Nature*.

[53] Barnes D. K. A. (2002). Biodiversity: invasions by marine life on plastic debris. *Nature*.

[54] Delfosse S., McGarry J., Morin T. (2010). *Ship Generated Waste Disposal in the Wider Caribbean Region*.

[55] Johnson L. S. (2008). *Cruise Ship Discharge Assessment Report*.

[56] Campani T., Baini M., Giannetti M. (2013). Presence of plastic debris in loggerhead turtle stranded along the Tuscany coasts of the Pelagos Sanctuary for Mediterranean Marine Mammals (Italy). *Marine Pollution Bulletin*.

[57] Smith S. D. A. (2012). Marine debris: a proximate threat to marine sustainability in Bootless Bay, Papua New Guinea. *Marine Pollution Bulletin*.

[58] Coombes E. G., Jones A. P. (2010). Assessing the impact of climate change on visitor behaviour and habitat use at the coast: a UK case study. *Global Environmental Change*.

[59] Todd P. A., Ong X., Chou L. M. (2010). Impacts of pollution on marine life in Southeast Asia. *Biodiversity and Conservation*.

[60] Defeo O., McLachlan A., Schoeman D. S. (2009). Threats to sandy beach ecosystems: a review. *Estuarine, Coastal and Shelf Science*.

[61] Kuo F.-J., Huang H.-W. (2014). Strategy for mitigation of marine debris: analysis of sources and composition of marine debris in northern Taiwan. *Marine Pollution Bulletin*.

[62] Carpenter A., Macgill S. M. (2005). The EU Directive on port reception facilities for ship-generated waste and cargo residues: the results of a second survey on the provision and uptake of facilities in North Sea ports. *Marine Pollution Bulletin*.

[63] Ball I. (1999). Port waste reception facilities in UK ports. *Marine Policy*.

[64] da Silva C. P. (2002). Beach carrying capacity assessment: how important is it?. *Journal of Coastal Research*.

[65] Hungerford H. R., Volk T. L. (1990). Changing learner behavior through environmental education. *The Journal of Environmental Education*.

[66] Butt N. (2007). The impact of cruise ship generated waste on home ports and ports of call: a study of Southampton. *Marine Policy*.

